# A Laser-Induced Graphene-Based Sensor Modified with CeO_2_ for Determination of Organophosphorus Pesticides with Improved Performance

**DOI:** 10.3390/s23239605

**Published:** 2023-12-04

**Authors:** Wenna Zhang, Qiu Sun, Xuelin Zhang, Weijian Yuan, Jianfeng Wu

**Affiliations:** 1 School of Chemistry and Chemical Engineering, Harbin Institute of Technology, Harbin 150001, China; 2 MEMS Center, Harbin Institute of Technology, Harbin 150001, China; 3 State Key Laboratory of Toxicology and Medical Countermeasures and Laboratory of Toxicant Analysis, Institute of Pharmacology and Toxicology, Academy of Military Medical Sciences, Beijing 100850, China

**Keywords:** laser-induced graphene, electrochemical sensor, organophosphorus pesticide, pralidoxime chloride, cerium dioxide

## Abstract

In this work, a flexible electrochemical sensor was developed for the detection of organophosphorus pesticides (OPs). To fabricate the sensor, graphene was generated in situ by laser-induced graphene (LIG) technology on a flexible substrate of polyimide (PI) film to form a three-electrode array, and pralidoxime (PAM) chloride was used as the probe molecule. CeO_2_ was used to modify the working electrode to improve the sensitivity of the sensor because of its electrocatalytic effect on the oxidation of PAM, and the Ag/AgCl reference electrode was prepared by the drop coating method. The effects of the laser power, laser scanning speed, and CeO_2_ modification on the electrochemical properties of the sensor were studied in detail. The results prove that the sensor has good repeatability, stability, and anti-interference ability, and it shows an excellent linear response in the chlorpyrifos concentration range from 1.4 × 10^−8^ M to 1.12 × 10^−7^ M with the detection limit of 7.01 × 10^−10^ M.

## 1. Introduction

Organophosphorus pesticides are widely used in agriculture, forestry, and animal husbandry to improve harvests due to their highly effective insecticidal, weeding, and sterilization capabilities [1]. However, excessive use of organophosphorus pesticides will lead to pesticide residues, which will pollute the environment and endanger food safety [2]. Moreover, the phosphate group carried by the organophosphorus pesticide will combine with cholinesterase to inhibit the catalytic hydrolysis of acetylcholine, which leads to the excessive accumulation of acetylcholine in the organism and thus causes neurotoxicity [3]. Therefore, it is of great significance to develop faster and more convenient pesticide residue detection methods to supervise the rational use of pesticides, ensure food safety management, and protect human life and health.

Traditional residue determination methods include high performance liquid chromatography [4], gas chromatography [5], and chromatography–mass spectrometry [6,7], which have the advantages of high efficiency determination and a wide determination range with adopting proper detectors, but the determination processes may be complex and tedious. Compared with traditional instrument detection methods, electrochemical sensors have the advantages of being simpler, smaller, and cheaper, with a fast response and high sensitivity [8]; so, they have become an effective method for the real-time detection of organophosphorus. For some OPs with active groups, such as methyl parathion (MP), which contains a nitrophenyl moiety, electrochemical signals can be directly measured to evaluate the concentration [9,10]. However, most organophosphorus do not have inherent redox activity. In order to detect organophosphorus without inherent redox activity, electrochemical sensors use the inhibition of organophosphorus on enzyme activity to detect organophosphorus pesticides [11,12,13]. However, complex detection conditions, including the temperature, pH value, and solvent type, will affect the detection activity of the enzymes. Moreover, the enzymes are unstable, costly, and cumbersome to operate [14]. These properties limit the wide application of enzymes in detecting Ops. Therefore, the study of non-enzyme electrochemical sensors is of great significance to detect pesticide residues more conveniently and sensitively.

**Scheme 1:**
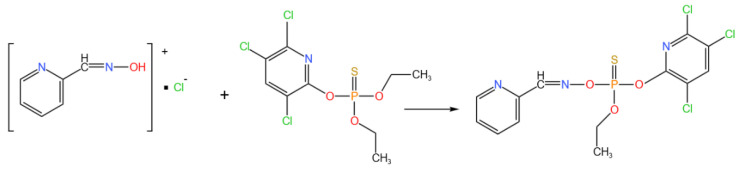
Illustration of the chemical reaction between PAM chloride and Ops.

Because oxime compounds that are commonly used as the antidote to organophosphorus can interact strongly with phosphate groups [15,16], as illustrated in Scheme 1, an electrochemical sensor with oxime compounds as probes has been designed to detect organophosphorus. By quantitatively analyzing the oxime oxidation current inhibited by Ops, the detection limits of 0.018 mM, 0.100 mM, and 0.215 mM can be obtained for chlorpyrifos, fenthion, and methyl parathion, respectively [17,18]. In our previous work, the composite materials of MWCNTs and CeO_2_ were prepared, and oxime compounds were used as probes to detect chlorpyrifos [19]. The sensor prepared has a low determination limit of 2.5 × 10^−9^ M, which proved that CeO_2_ nanoparticles can improve the performance of the oxime-based sensor by effectively catalyzing the oxime oxidation reaction. Despite of the success of oxime-based sensors in OP determination, it is still necessary to explore some new strategies of electrode design to improve their determination ability.

Recently, laser-induced graphene (LIG) technology has received great attention as an effective way to fabricate some flexible and portable electronic devices [20,21,22,23,24]. This technology uses the photothermal effect caused by laser irradiation to break the C=O, C-C, and N=C bonds to form C-C bonds and thus promote the conversion of sp^3^ carbon atoms to sp^2^ carbon atoms to form the porous graphene layer in situ on the substrates of PI, PDMS, and so on [25,26]. By avoiding the complex and time-consuming processes of graphene synthesis and electrode modification, LIG technology is much faster, simpler, and easier compared with the traditional preparation method of a graphene-based electrode [27,28]. Although LIG technology has been widely and successfully used to fabricate different kinds of electrochemical sensors to detect ascorbic acid, dopamine, uric acid, and heavy metal ions, it has not been used in the fabrication of oxime-based electrochemical sensors.

In this work, an oxime-based sensor was constructed on flexible PI surfaces using laser engraving technology, and the effects of the laser power and laser scanning speed on the electrochemical properties of the sensor were investigated. By modifying the sensor with CeO_2_ nanoparticles, a low-cost, portable, and flexible sensor was developed to detect OPs with improved sensitivity. The electrochemical properties of the fabricated LIG sensors were characterized by CV and DPV in K_3_[Fe(CN)_6_] solution and PAM chloride solution. In addition, the repeatability, stability, and anti-interference of the sensor were also tested to illustrate the practicability of the flexible electrochemical sensor. Considering the miniaturization and portability of the electrochemical equipment [29], the proposed sensor would have potential practical application in the determination of OPs.

## 2. Materials and Methods

### 2.1. Reagents and Instruments

The PI film (125 μm thickness) and Kapton tape (125 μm thickness) were purchased from Dupont TM (Hayward, CA, USA). Cerium dioxide, phosphoric acid, sodium hydroxide, chlorpyrifos, and phosphorus chlorolysis were ordered from Aladdin (Shanghai, China). Silver/silver chloride paste for reference electrode printing was ordered from Sigma Aldrich (Shanghai, China).

PI was induced into graphene by a CO_2_ laser in an argon atmosphere. The electrochemical response test was conducted in a CHI760D electrochemical workstation (Shanghai Chenhua Co., Ltd., Shanghai, China). The scanning electron microscope (SEM, SU8010, Tokyo, Japan) and transmission electron microscopy (TEM, Tecnai 20, Amsterdam, The Netherlands) were used to obtain the morphology of LIG. The LabRAM HR Evolution Raman microscope system (Horiba Jobin Yvon) was used to measure and obtain the Raman spectrum of LIG. The electrode was dried in a WS70-1 infrared drying furnace (Shanghai Gaozhi Company, Shanghai, China). The ultrasonic dispersion of samples was carried out in the SCIENTZ-950E ultrasonic cell pulverizer (China Ningbo Sanda Technology Co., Ltd., Ningbo, China).

### 2.2. Preparation of the LIG Electrode

**Figure 1:**
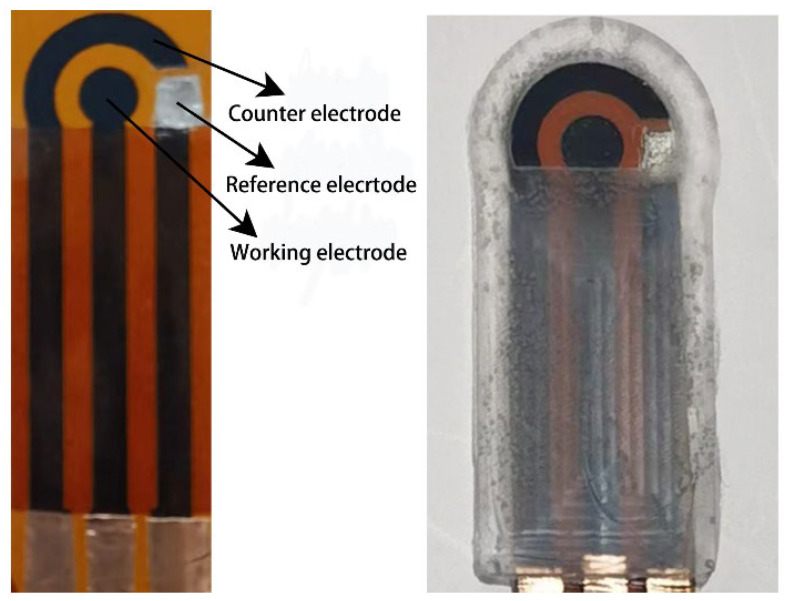
The images of the sensor fabricated by the LIG technique.

Before conducting the laser treatment, the PI film was cleaned sequentially by ethanol and deionized water and then dried with nitrogen (N_2_). To prevent the thermal deformation during the laser process, Kapton tape was pasted on the back of the PI film to form a double-layer substrate. A CO_2_ laser with different scan rates and laser powers was used to directly convert the PI into 3D porous graphene, where the working area is a circle with 3 mm in diameter, and the lead wire is encapsulated with polyimide tape to avoid its contact with the electrolyte solution. Three copper strips were pasted on the wear of the lead wire to facilitate the connection with the electrochemical workstation. Finally, Ag/AgCl slurry was coated on the reference electrode and baked in an oven at 60 °C for 30 min. The images of the as-fabricated sensor are shown in Figure 1.

### 2.3. Preparation of the Modified Electrode

CeO_2_ dispersion was prepared by ultrasonic dispersion and used as the modified material of the electrode. First, 4 mg of CeO_2_ NPs was dispersed in 2 mL ethanol and then ultrasonically dispersed in an ultrasonic cell grinder for 5 min; finally, a 2 mg/mL CeO_2_ NP suspension was obtained, and then 1 mg/mL Nafion was dropped as the fixative. CeO_2_/LIG was prepared by dropping 4 μL of modified material on the surface of the LIG electrode and then drying in an infrared fast drying furnace.

### 2.4. Analysis Program

The prepared three electrodes were inserted into a phosphate buffer solution (30 mL, 0.1 M) containing PAM chloride (0.1 mM). The DPV was used to scan in the potential range of 0.1 to 0.9v. The step was 4 mV, the amplitude was 50 mV, the pulse width was 0.08 s, the sample width was 0.004 s, the pulse period was 0.8 s, and the quiet time was 180 s. It was used to characterize the electrochemical performance of CeO_2_/LIG on PAM chloride and chlorpyrifos.

## 3. Results

### 3.1. Basic Characteristics of the LIG Electrode

**Figure 2:**
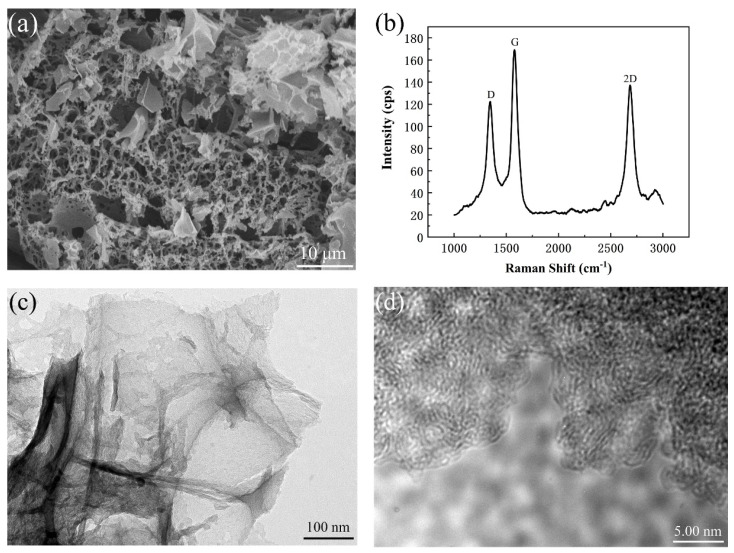
SEM images of LIG (**a**); Raman spectra of LIG (**b**); TEM images of LIG at different resolutions (**c**,**d**).

The CO_2_ laser direct writing device was used to prepare the LIG with the laser power of 5.01 W at a speed of 2.33 cm s^−1^, and the typical structure of the as-formed porous graphene layer was characterized by SEM and Raman spectra. As shown in Figure 2a, abundant layered porous structures were observed, which were formed during the graphitization process by the photothermal transformation of laser irradiation. And three very obvious graphene characteristic peaks can be seen from the Raman spectrum shown in Figure 2b, namely, the D peak at 1340 cm^−1^, the G peak at 1590 cm^−1^, and the 2D peak at 2670 cm^−1^. Among them, the formation of the G peak is caused by the in-plane vibration of the sp^2^ carbon atom, while the D peak is caused by the defect of graphene itself or the bent sp^2^ carbon bond (the bent graphene layer), which confirms the formation of a graphene structure [30]. Through calculation, the intensity ratio of G peak and D peak is about: *I*_D_/*I*_G_ = 0.98, which indicates that the LIG is highly crystalline.

In addition, from the low-resolution image of the LIG shown in Figure 2c, it can be observed that graphene presents a staggered stack structure of thin patches. After further enlarging the local area of the graphene thin-layer surface, it can be observed from the high-resolution image in Figure 2d that graphene presents rich fold shapes and ripple structures. The appearance of this structure is caused by the instantaneous thermal shock and rapid thermal expansion when laser induced graphene is generated [31,32]. The above results show that the laser induced graphene is a few-layered graphene with a layered structure rather than graphite.

### 3.2. Effects of the Laser Parameters on the Electrochemical Properties of LIG

**Figure 3:**
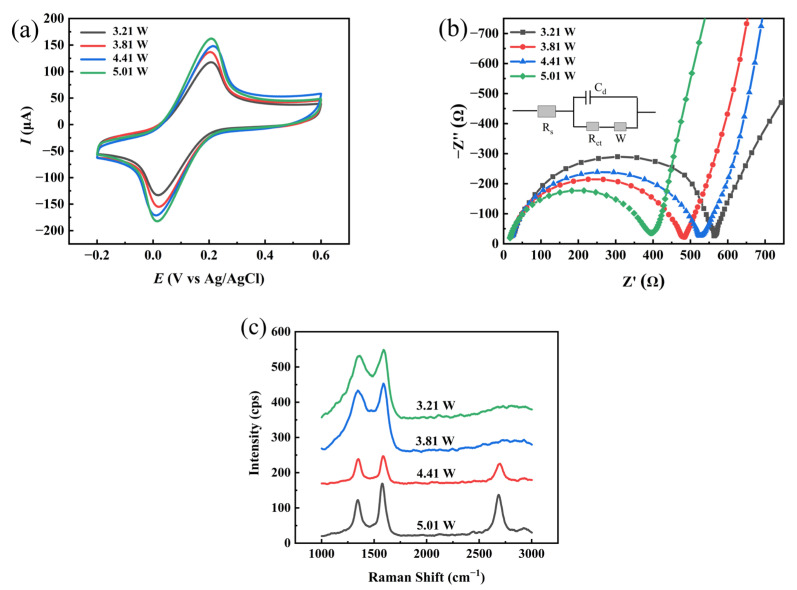
CV curves of LIG electrodes prepared by different laser powers with a laser scan speed of 2.33 cm s^−1^ in a 0.1 M KCl solution containing 5.0 mM K_3_[Fe(CN)_6_] (**a**); EIS plots of electrodes under different laser powers with a laser scan speed of 2.33 cm s^−1^ in a 0.1 M PBS solution containing 0.1 mM PAMCl (**b**); Raman spectra of LIG electrodes prepared by different laser powers with a laser scan speed of 2.33 cm s^−1^ (**c**).

The laser powers of 3.21 W, 3.81 W, 4.41 W, and 5.01 W were separately adopted to prepare four electrodes at the same laser speed of 2.33 cm s^−1^. In order to characterize the electrochemical properties of the above electrodes, 5 mM K_3_[Fe(CN)_6_] was used as the electrochemical probe to perform the CV tests in 0.1 M KCl solution, and the results are shown in Figure 3a. It can be seen that there is a pair of redox peaks in the CV curve of the LIG electrode. With the increase in the laser power, the peak current of the electrode gradually increases. When the laser power is 5.01 W, the as-fabricated LIG electrode gives the maximum peak current of 121 μA.

In order to further verify the effect of the laser power on the electrochemical characteristics of the electrode, LIG prepared under different laser powers underwent EIS testing in an electrolyte solution containing 0.1 mM PAMCl and 0.1 M PBS. As shown in Figure 3b, a single semicircle and a line appear in Nyquist curves, which correspond to the electron transfer process and the diffusion process, respectively. As is well accepted, the semicircle diameter in Nyquist curves is proportional to the charge transfer resistance of the electrochemical oxidation reaction of PAMCl, *R*_ct_, which means that the *R*_ct_ value can be used to evaluate the property of the electrode kinetics. It can be seen that as the laser power increases, the *R*_ct_ value of the LIG gradually decreases. When the laser power is 5.01 W, the minimum *R*_ct_ value can be obtained, which proves that the LIG electrode fabricated at 5.01 W has the best electrochemical activity toward PAMCl oxidation.

To illustrate how the electrochemical performance of the LIG electrodes are affected by the laser power, Raman spectra were conducted and the results are shown in Figure 3c. The Raman curves reveal that 2D peaks appear when the laser power is 5.01 W and 4.41 W, which proves that the porous graphene has come into being. But the 2D peaks disappear when the laser power is reduced to 3.81 W, which is related to the fact that the laser power does not reach the lowest energy for graphene generation and only graphite is formed. In addition, it is found that the *I*_D_/*I*_G_ ratio of the LIG decreases with the increase of laser power, indicating that the quality of the generated graphene is improved, which is consistent with the result that the peak current of LIG electrode gradually increases with the laser power [33,34].

**Figure 4:**
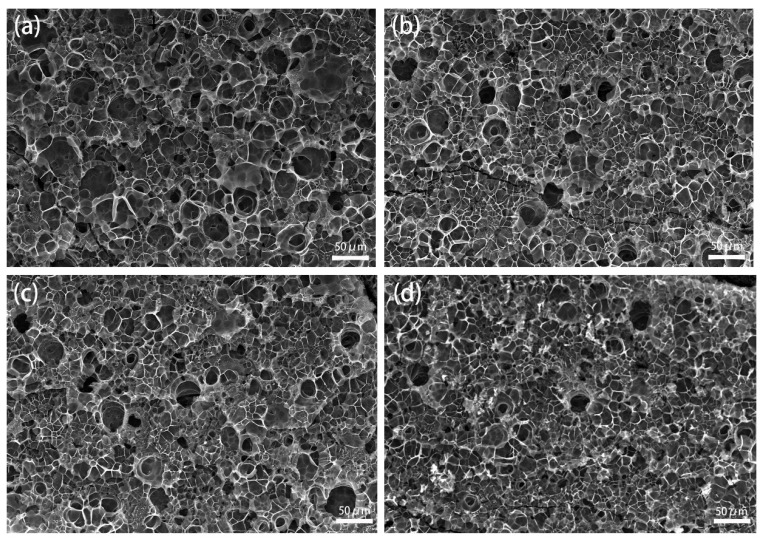
SEM images of the LIG electrodes prepared by different laser powers of 5.01 W (**a**), 4.41 W (**b**), 3.81 W (**c**), and 3.21 W (**d**) with a laser scan speed of 2.33 cm s^−1^.

Moreover, the surface morphologies of the LIG electrodes were also characterized by SEM measurement, as shown in Figure 4. It can be seen clearly that the electrode has a typical porous structure. At the laser power of 3.21 W, the pore size of the electrode is relatively small. With the laser power increased to 3.81 W and 4.41 W, the pore sizes of the two electrodes increase slightly, but it seems that there is no obvious difference between the two electrodes. When the laser power is increased to 5.01 W, the pore size of the electrode increases obviously. These results indicate that the LIG porosity increases with the laser power; so, the specific surface area increases, which is beneficial for electron transfer [35]. However, when the laser power is larger than 5.01 W, the LIG peels off from the PI surface. This should be due to the formation of more layered porous graphene at high laser power, and the excess energy will cause the pore edge to be burnt into fragments and fall off [31,36].

**Figure 5:**
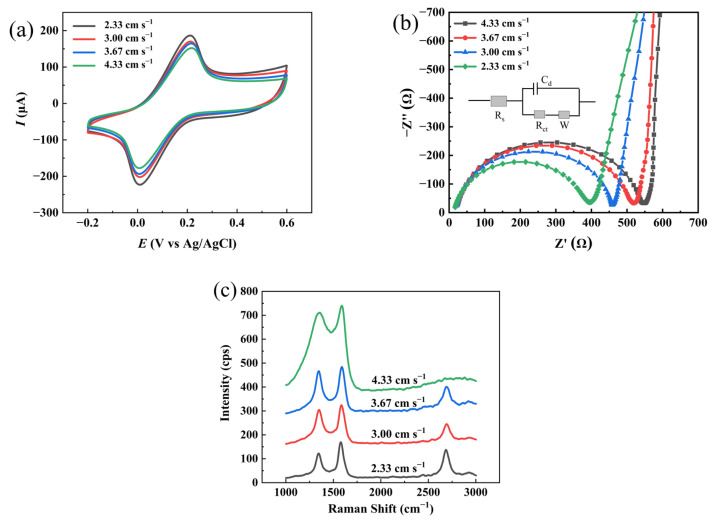
CV curves of LIG electrodes prepared by different laser scan speeds with a power of 5.01 W in a 0.1 M KCl solution containing 5.0 mM K_3_[Fe(CN)_6_] (**a**); EIS plots of electrodes under different laser scan speeds with a power of 5.01 W in a 0.1 M PBS solution containing 0.1 mM PAMCl (**b**); Raman of LIG electrodes prepared by different laser scan speeds with a power of 5.01 W (**c**).

Then, the CO_2_ laser scanning speeds of 4.33 cm s^−1^, 3.67 cm s^−1^, 3.00 cm s^−1^, and 2.33 cm s^−1^ were used to fabricate the electrodes with the laser power being set as 5.01 W. To characterize the effects of the scanning speed on the electrochemical response, the LIG electrodes were subjected to CV measurements in 0.1 M KCl solution containing 5.0 mM K_3_[Fe(CN)_6_] It can be seen from Figure 5a that as the laser scanning speed decreases, the peak current of the electrode gradually increases, and the maximum peak current is achieved at the laser scanning speed of 2.33 cm s^−1^. The EIS measurements were also conducted in 0.1 M PBS solution containing 0.1 mM PAMCl. The Nyquist curves shown in Figure 5b also reveal that the *R*_ct_ value of the LIG electrode gradually decreases with the laser scanning speed, and the electrode prepared at the laser scanning speed of 2.33 cm s^−1^ has the smallest *R*_ct_ value, which corresponds well with those of the CV measurements.

**Figure 6:**
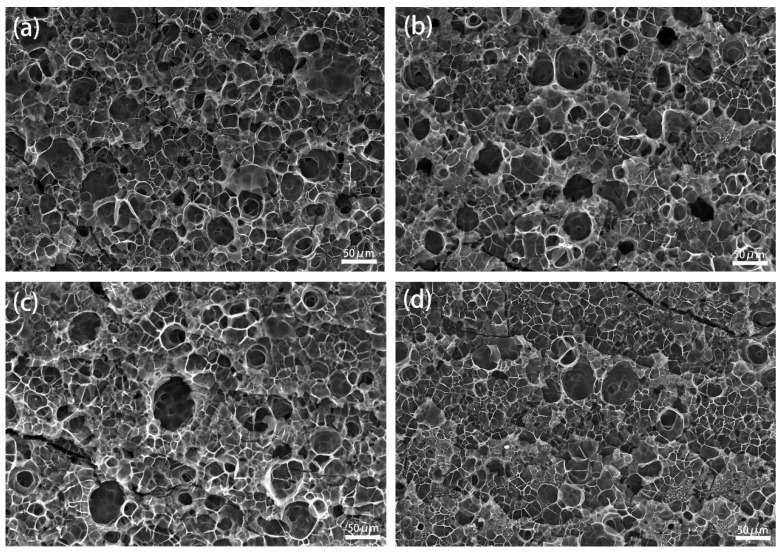
(**a**–**d**) SEM images of the LIG electrodes prepared by different laser scan speeds (2.33 cm s^−1^, 3.00 cm s^−1^, 3.67 cm s^−1^, 4.33 cm s^−1^) with a power of 5.01 W.

According to the Raman spectra shown in Figure 5c, when the laser scanning speeds are 2.33 cm s^−1^, 3.00 cm s^−1^, and 3.67 cm s^−1^, the observed 2D peaks indicate the formation of the porous graphene layer. However, when the laser scanning speed is increased to 4.33 cm/s, the 2D peaks disappear. This is because the laser scanning speed is too fast, and the energy retention time is too short, which means the amorphous carbon cannot be converted into graphene in time. With the decrease in the laser scanning speed, the *I*_D_/*I*_G_ ratio of the graphene decreases, indicating the improved quality of the graphene. Moreover, as shown in Figure 6a–d, the porosity of the LIG also increases with a decrease in the laser scanning speed. However, when the laser speed is less than 2.33 cm s^−1^, the LIG electrode appears mottled, and the porous graphene peels off easily, which should be ascribed to the fact that the long laser residence time results in excessive combustion at high temperature and the collapse of pore structure. Therefore, the laser power of 5.01 W and the laser speed of 2.33 cm s^−1^ were selected as the optimal parameters for preparing the LIG electrodes [37].

### 3.3. Effects of the CeO_2_ Modification on the Electrochemical Properties of the LIG-Based Sensor

**Figure 7:**
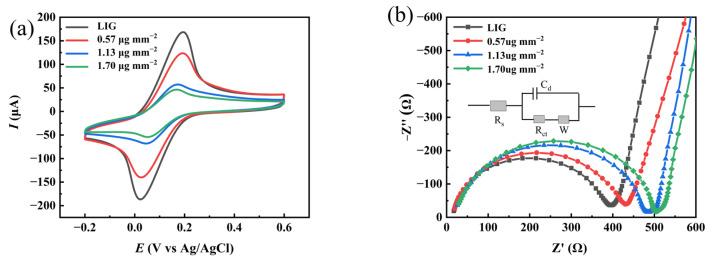
CV curves (**a**) and EIS plots (**b**) of the various electrodes in a 0.1 M PBS (pH 7) containing 0.1 mM PAM chloride at the scan rate of 0.1 V/s.

As illustrated in our previous research work, CeO_2_ nanoparticles show great catalytic activity toward PAMCl electrochemical oxidation, which can improve the sensitivity of the sensor for OP detection to a great extent. To optimize the loading of CeO_2_ nanoparticles on the LIG electrode, four different electrodes were prepared, namely a bare LIG, 0.57 μg/mm^2^ CeO_2_/LIG, 1.13 μg/mm^2^ CeO_2_/LIG, and 1.70 μg/mm^2^ CeO_2_/LIG. In order to characterize the electrochemical properties of the above electrodes, CV measurements were carried out in a 0.1 M KCl solution containing 5 mM K_3_[Fe(CN)_6_], and the results are shown in Figure 7a, where a pair of redox peaks can be observed. For the bare LIG, the peak currents are 121 µA (*I*_pa_) and 122 µA (*I*_pc_), respectively. With the increase in the CeO_2_ amount, the peak currents of the electrode decrease significantly, which is attributed to the fact that CeO_2_ does not conduct electricity, preventing the transfer of electrons on the electrode surface.

In order to further verify the electrochemical performance of the modified electrodes, electrochemical impedance tests were conducted on the four types of modified electrodes. The impedance curves of the electrodes in an electrolyte solution containing 0.1 mmol L^−1^ PAMCl and 0.1 mol L^−1^ PBS are shown in Figure 7b. The electrochemical impedance value of LIG was observed to be the smallest, indicating that the electrode has the best conductivity. When CeO_2_ is modified on the working electrode surface of LIG, it is found that the impedance value of the electrodes increases significantly, and with the increase in the CeO_2_ modification amount, the impedance value of the electrode increases, which proves that CeO_2_ does have the characteristic of blocking electron transfer.

**Figure 8:**
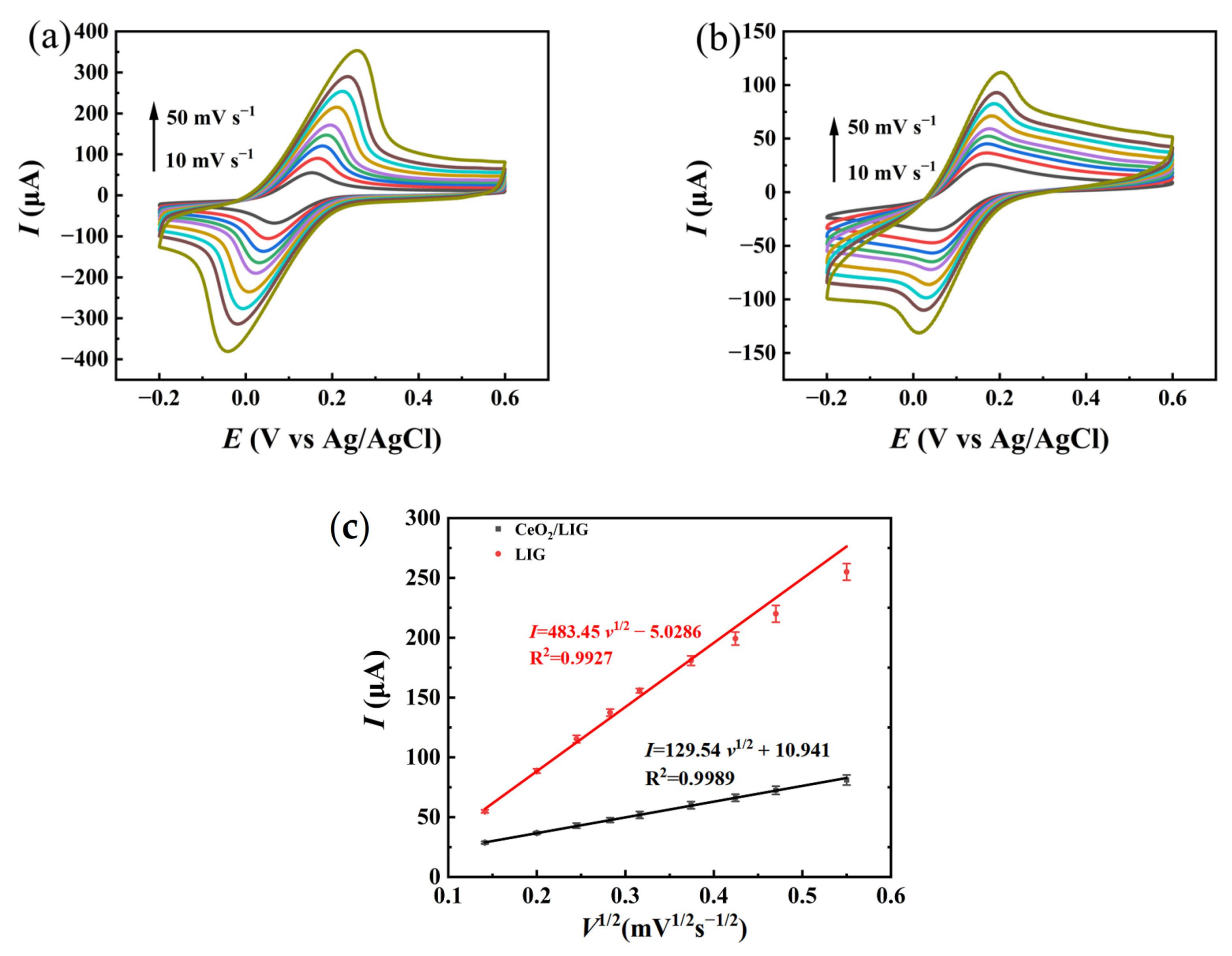
CV curves of the LIG (**a**) and 1.13 μg/mm^2^ CeO_2_/LIG (**b**) with varying scan rate from 10 to 50 mV/s (as illustrated by the arrows) in 0.1 M KCl containing 5 mM K_3_[Fe(CN)_6_]. Linear relationship between IP and *ν*^1/2^ for various electrodes (**c**).

In order to quantitatively illustrate the effect of the CeO_2_ modification on the LIG electrode, bare LIG and 1.13 μg/mm^2^ CeO_2_/LIG electrodes were subject to the measurement of a CV test at different scanning rates in the above electrolyte solutions, and the CV curves are shown in Figure 8a,b. When the scanning rate is from 10 mV/s to 50 mV/s, the oxidation peak currents of the two electrodes have a linear relationship with the square root of the scanning rate (Figure 8c). And the electroactive area of the electrode can be calculated according to the Randles Sevcik model [38].

*I*_p_*=* 2.69 *×* 10^5^*n*^3/2^*AD*_0_^1/2^*v*^1/2^*C,* (1)

where *n* is the number of electrons transferred in the redox process, *A* is the electroactive area of the electrode, *D*_0_ is the diffusion coefficient (6.73 × 10^−6^ cm^2^ s^−1^), *v* is the scan rate, and *C* is the concentration of the probe in solution (5 × 10^−6^ mol cm^−3^). According to the above formula, the electroactive area of the bare LIG is 0.149 cm^2^, which is much larger than its physical area (about 0.07 cm^2^). The electroactive area of the CeO_2_/LIG is 0.038 cm^2^. Obviously, the modification of CeO_2_ on the electrode surface impedes the electron transfer on the electrode surface and reduces the electrochemical active area, indicating that CeO_2_ has successfully modified the electrode surface.

**Figure 9:**
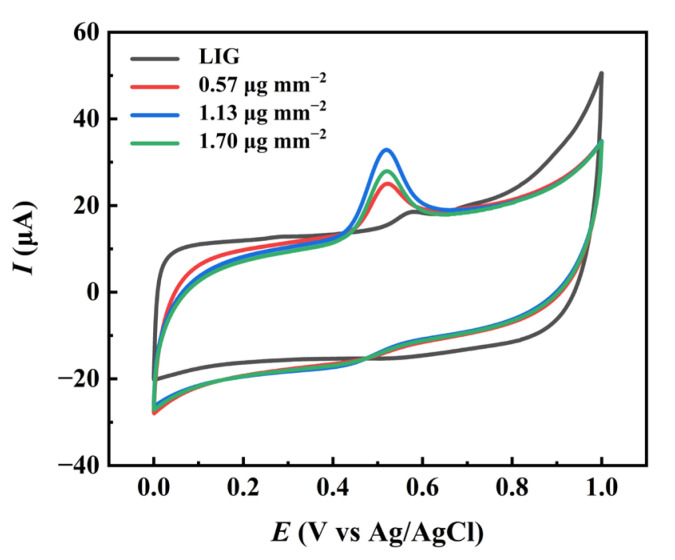
CV of the various electrodes in 0.1 M PBS (pH 7) containing 0.1 mM PAM chloride.

In order to investigate the differences in the electrochemical response of electrodes with different CeO_2_ modified in the PAMCl oxidation reaction, CV measurements were performed on electrodes with different CeO_2_ modification amounts in 0.1 M PBS (pH 7) containing 0.1 mM PAM chloride, as shown in Figure 9. It can be seen that the potential of the LIG electrode shows a small oxidation peak. When CeO_2_ was modified onto the surface of the LIG electrodes, it was found that the peak currents of the electrodes significantly increased. Moreover, it can be seen that as the CeO_2_ modification amount increases, the peak current gradually increases. This is mainly because CeO_2_ can catalyze the oxidation reaction of PAMCl itself, proving that CeO_2_ has high catalytic activity for oxime oxidation. The more CeO_2_ is modified, the stronger the electrocatalytic effect of CeO_2_/LIG electrode toward the oxidation of oxime compounds; so, the peak current gradually increases.

However, the peak current of PAM chloride was found to reach its peak value of 18.61 µA when the CeO_2_ droplet coating amount was 1.13 μg mm^−2^. The peak current shows a gradually decreasing trend with the subsequent increase in the droplet coating amount. This should be due to the fact that a too thick modified material can hinder the electron transfer instead and deteriorate the electrochemical performance. Therefore, 1.13 μg mm^−2^ CeO_2_ nanomaterial was selected as the optimal amount for the next experiments.

**Figure 10:**
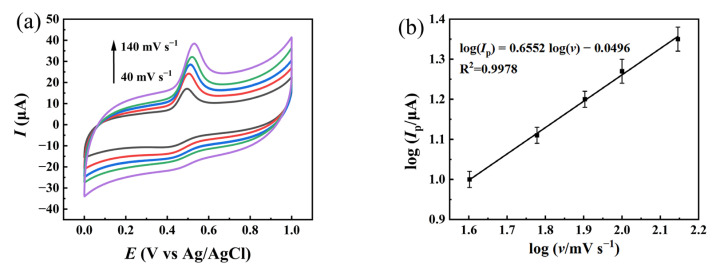
CVs of the 1.13 μg/mm^2^ CeO_2_/LIG varying the scan rate from 40 to 140 mV/s in 0.1 M PBS (pH 7) containing 0.1 mM PAM chloride (**a**); Linear relationship between log(*I*_p_) and log(*ν*) for electrodes with scanning rates of 40 to 140 mV/s (**b**).

To obtain further insight into the oxidation reaction of PAMCl on the electrode, the CV curves of 1.13 μg/mm^2^ CeO_2_/LIG electrode at different scanning rates in a 0.1 M PBS solution containing 0.1 mM PAM chloride are shown in Figure 10a,b. When the scanning rate is 10–50 mV/s, the logarithm of peak current and the logarithm of scanning rate show a linear relationship. The relationship between log (*I*_p_) and log (*v*) can be described as a linear regression equation [39].

log(*I*_p_) (µA) = −0.43 + 0.80 log(*v*) (mV/s) (2)

In the above model, different values of the slope between log(*I*_p_) and log(*v*) represent different control process of the reaction on the electrode. When the value is 0.5, the reaction on the electrode is controlled by a diffusion process. When the value is 1, the reaction on the electrode is controlled by an adsorption process. When the value is between 0.5 and 1, the reaction on the electrode is controlled by both adsorption and diffusion processes. In the linear regression equation, the slope is 0.63; so, the reaction of PAMCl oxidation on the electrode is controlled by both adsorption and diffusion processes.

**Figure 11:**
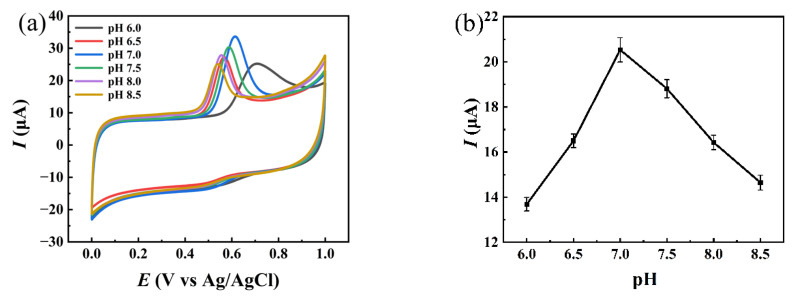
The corresponding relationship between the peak current and pH (**a**,**b**).

In order to investigate the effect of the pH value on the peak current, the electrochemical response of PAM chloride in different pH solutions was measured. Since the surface PAM chloride was studied to be suitable for neutral or alkaline systems, the pH range of 6 to 8.5 was taken as the test range [40]. It can be seen from Figure 11a,b that the electrochemical activity of PAMCl is inhibited to some extent in acidic and alkaline solutions. Therefore, the optimal pH of the electrolyte solution is 7.

### 3.4. Analytical Performance of the LIG-Based Sensor for Chlorpyrifos Determination

**Figure 12:**
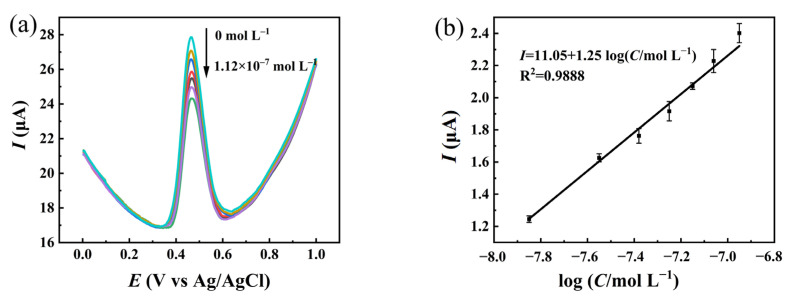
DPV curves using 0.1 mM PAM chloride as the electrochemical probe in 0.1 M PBS (pH 7) for the detection of chlorpyrifos with the concentration changing from 0 to 1.12 × 10^−7^ M (as illustrated by the arrow) (**a**); calibration plot for detecting chlorpyrifos (**b**).

After optimizing the experimental conditions, the performance of the LIG-based sensor was evaluated by conducting the DPV measurements in different concentrations of chlorpyrifos with PAMCl as an electrochemical probe, and the DPV curves are shown in Figure 12a, where the peak currents decrease with the concentration of chlorpyrifos. It is found that there is a linear relationship between the logarithm of the added chlorpyrifos and the inhibition rate of the peak current in the concentration range of 1.4 × 10^−8^ to 1.12 × 10^−7^ M, as shown in Figure 12b, which can be described by the following linear regression Equation (3).

*I*(μA) = 11.05 + 1.25log*C* (3)

The detection limit of the prepared electrochemical sensor is calculated by the formula DL = 3 × SDB/sensitivity. SDB represents the standard deviation obtained by 10 parallel determinations in blank solution. Finally, the detection limit is calculated to be 7.01 × 10^−10^ M (S/N = 3).

To illustrate the advantage of the as-fabricated sensor, the detection limit of chlorpyrifos was compared with those of other sensors that also adopt PAM chloride as the electrochemical probe, as shown in Table 1. It can be found that the sensor fabricated in this work has a lower detection limit because of the unique property of the LIG. At the same time, compared with most sensors that are reported for chlorpyrifos detection in the literatures, the as-fabricated sensor also presents a much lower detection limit. This confirms that the study provides a cheap, sensitive, and portable method for the determination of chlorpyrifos in organophosphorus pesticides.

**Figure 13:**
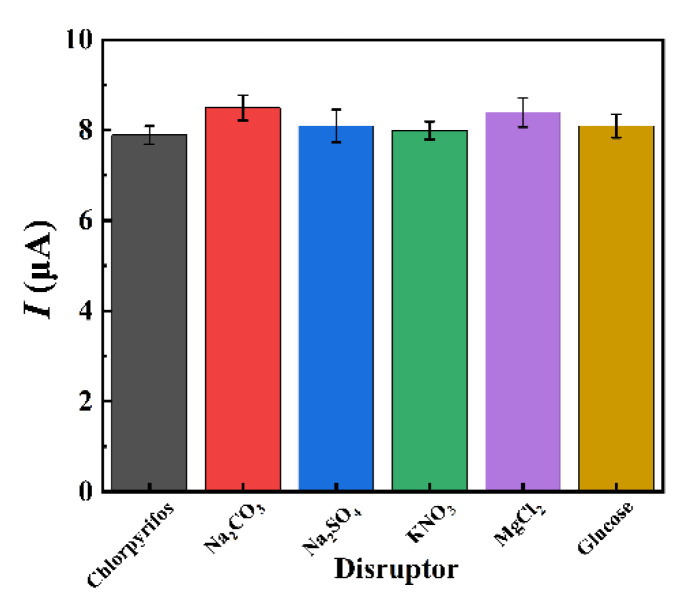
Study on the anti-interference ability of 1.13 μg/mm^2^ CeO_2_/LIG.

To conduct the anti-interference measurement, common interfering substances, such as Na_2_CO_3_, Na_2_SO_4_, KNO_3_, MgCl_2_, and glucose at a concentration of 100 times that of chlorpyrifos, were added to the solution containing 0.1 mM PAMCl and 1 × 10^−7^ M chlorpyrifos, and then the DPV measurements were conducted, as shown in Figure 13. It can be seen that the peak current does not change significantly with a variation less than 6.8%. The experiment shows that the sensor has excellent anti-interference performance in the determination of chlorpyrifos.

In order to evaluate the reproducibility of the electrochemical sensor, the same steps were taken to prepare 10 electrodes. After adding chlorpyrifos of the same concentration to the solution containing 0.1 mM PAMCl, DPV measurements were performed on the 10 electrodes to obtain the peak currents. After calculation, the relative standard deviation (RSD) of the electrochemical response was 5.8%. In order to evaluate the stability of the electrode, the working electrode was used to conduct continuous DPV tests in the above solution, and the results show that the relative standard deviation of peak currents was 6.9%. The above results show that the electrode has good reproducibility and stability in the detection of chlorpyrifos.

Finally, two types of vegetable extracts were adopted to conduct the recovery experiments to evaluate the effectiveness of the LIG sensor for the determination of chlorpyrifos in real samples. The test solution was prepared by mixing a certain amount of lettuce and spinach juice with chlorpyrifos, and the results are shown in Table 2. In the spinach juice, the recovery rates of the spiked chlorpyrifos ranged from 96.97% to 104.2%, while in the lettuce juice, the recovery rates ranged from 95.97% to 103.67%. The above results confirm the reliable determination of chlorpyrifos in real samples by the as-fabricated LIG sensor.

## 4. Conclusions

Using laser-induced graphene technology, a graphene flexible electrode was prepared by laser direct writing on the PI surface. Using PAM chloride as the electrochemical probe, CeO2 NP was modified on the electrode to catalyze oxime hydroxy oxidation, and a flexible electrochemical sensor was prepared to detect OP without electrochemical redox activity. The results show that the graphene prepared by LIG technology has excellent electron transfer rate. A high laser power will increase the porosity and promote the electron transfer, but excessive laser power will lead to excessive exhaust gas and structure fracture. A low scanning speed will increase the graphitization of the material and enhance the conductivity. Too low a scanning speed will lead to excessive combustion at high temperature. The prepared graphene electrode was used to detect organic phosphorus. It was found that the flexible sensor showed an excellent electrochemical response, and the modified CeO_2_ NPs played an obvious catalytic role in oxime oxidation. Under the optimized experimental conditions (pH = 7), the inhibition rate of the PAM chloride peak current has a good linear response relationship with the logarithm of chlorpyrifos concentration, with a range of 1.4 × 10^−8^ M to 1.12 × 10^−7^ M, and the detection limit is 7.01 × 10^−10^ M. The response of the sensor to chlorpyrifos was measured 10 consecutive times, and it was found that the repeatability was good, with an RSD of 5.8%. Moreover, the influence of interfering substances on the sensor was detected, and it was found that the anti-interference ability of the sensor was good. Therefore, the flexible electrochemical sensor has high practical value, convenient preparation, high sensitivity, strong applicability, and low price and is expected to be used in wearable sensing and portable monitoring fields.

## Figures and Tables

**Table 1:** Comparison with other sensors for the determination of chlorpyrifos.

Electrode Material	Linear Range	LOD	Reference
GO/g-C_3_N_4_/GCE	0.8 nM−0.01 μM	0.82 nM	[10]
AChE/CS-GO/GO/CNFs	25–1000 nM	2.2 nM	[41]
CeO_2_/MWCNTs/GCE	0.05 μM^−1^ μM	2.5 nM	[19]
CuO/ITO (PAM)	0.01–0.16 μM	1.6 nM	[42]
MIPs/GCE	0.0001–10 μM	4.1 nM	[17]
GCE (PAMCl)	25 nM−13.5 μM	18 nM	[43]
CeO_2_/LIG (PAMCl)	0.014–0.112 μM	0.7 nM	This work

**Table 2:** Results of recovery experiments in real samples containing chlorpyrifos.

Sample	Taken (nM)	Found (nM)	Recovery (%)	RSD (%)
Spinach	30	31.26 ± 1.00	104.2 ± 3.33	3.20
	100	96.97 ± 3.2	96.97 ± 3.19	3.30
Lettuce	30	28.79 ± 0.58	95.97 ± 1.94	2.01
	100	103.67 ± 2.39	103.67 ± 2.39	2.31

## Data Availability

The data that support the findings of this study are available upon reasonable request.
